# Supplementary material for: Observation of E-cadherin adherens junction dynamics with metal-induced energy transfer imaging and spectroscopy
Source: Commun Biol. 2024 Nov 30;7:1596. doi: 10.1038/s42003-024-07281-4 (PMC11606943; doi:10.1038/s42003-024-07281-4)
Supplement: Supplementary file 2 — Supplementary Information [file 42003_2024_7281_MOESM2_ESM.pdf]

Supplementary Information:

Observation of E-cadherin Adherens Junctions Variation with Metal-induced Energy Transfer Imaging/Spectroscopy

Tao Chen, Narain Karedla, Jörg Enderlein

Tap Chen
E-mail: tao.chen@phys.uni-goettingen.de;
Jörg Enderlein
E-mail: jenderl@gwdg.de

Supplementary Note 1. Materials

Lipids 1-stearoyl-2-oleoyl-sn-glycero-3-phosphocholine (SOPC), 1,2-dipalmitoyl-sn-glycero-3-phosphocholine (DPPC), 1,2-dioleoyl-sn-glycero-3-[(N-(5-amino-1-carboxypentyl)iminodiacetic acid)succinyl] (nickel salt) (DOGS), 1,2-distearoyl-sn-glycero-3-phosphoethanolamine-N-[methoxy(polyethylene glycol)-2000] (ammonium salt) (PEG2000-DOPE), 1,2-dioleoyl-sn-glycero-3-phosphoethanolamine-N-(cap biotinyl) (sodium salt) (DOPE-cap-Biotin), and 1-palmitoyl-2-6-[(7-nitro-2-1,3-benzoxadiazol-4-yl)amino]hexanoyl-sn-glycero-3-phosphocholine (NBD-PC) were purchased from Avanti Polar Lipids and diluted at 10 mg/mL with chloroform for stock solution. Atto655 labeled 1,2-Dipalmitoyl-sn-glycero-3-phosphoethanolamine (DPPE) (Atto655-DPPE) was purchased from Atto-TEC and dissolved in chloroform at 1 mg/mL for stock solution.

Proteins, neutravidin, and E-Cadherin-Fc chimera were used following the manufacturer's instructions. The neutravidin protein (Thermo Scientific™) was reconstituted with ultrapure water and then diluted to 1 mg/mL with PBS (140 mM NaCl, 3 mM KCl, 10 mM Na_2HPO_4, 2mM KH_2PO_4, pH 7.4). The recombinant human E-cadherin-Fc chimera (referred to as E-cad, Biolegend) was reconstituted with PBS at 100 μg/mL and stored at -80°C.

Supplementary Note 2. Suppression of diffusion signal in FCS by using high dye concentration

To mitigate the influence of lipid diffusion on our fluorescence intensity-dependent measurements, we employed a high dye concentration (1 mol%) of DPPE-Atto655 in the lipid bilayer. This strategy, recommended by Monzel et al. (1), effectively minimizes diffusion-related signals. To confirm that this dye concentration adequately eliminates fluorescecne intensity fluctuations induced by diffusion, we conducted fluorescence correlation spectroscopy (FCS) experiments on supported lipid bilayers (SLBs) with varying dye concentrations.

In Supplementary Figure 2a, the autocorrelation function (ACF) obtained from an SLB with a dye concentration of 0.001 mol% reveals modulation in the correlation curve due to the diffusion of fluorophores through the confocal volume. In contrast, when the dye concentration in the SLB is increased to 1 mol% (Supplementary Figure 2b, black curve), the FCS signal diminishes, and the ACF flattens, indicating effective suppression of diffusion-related intensity fluctuations.

Furthermore, we performed FCS measurements on a fluctuating membrane by immersing a highly labeled GUV (1 mol%) in a hypertonic solution (300 mOsm/L, PBS). In this scenario, the GUV becomes floppy, and its membrane undergoes significant bending fluctuations. As anticipated, we observed a strong correlation decay in the ACF for such a deflated GUV (Supplementary Figure 2b red curve). Due to the high dye labeling, this signal is devoid of any contributions from lateral dye diffusion, and the correlation amplitude solely results from axial displacements of the membrane.

Supplementary Note 3. Fluorescence Recovery after Photobleaching (FRAP) measurements

To investigate the diffusion dynamics of NBD-PC-labeled lipids in both solid-supported phospholipid bilayers (SLBs) of 1-palmitoyl-2-oleoyl-sn-glycero-3-phosphocholine (SOPC) and

1,2-dioleoyl-sn-glycero-3-phosphocholine (DPPC), fluorescence recovery after photobleaching (FRAP) measurements were employed. Both SOPC and DPPC SLBs were prepared using the Langmuir-Blodgett (LB)-Langmuir-Schaefer (LS) method, with 1 mol% NBD-PC, 2 mol% polyethylene glycol (PEG) 2000-doped DOPE, and 5 mol% DOGS-NTA incorporated into the distal layer of the bilayer. A 488 nm laser beam was used to photobleach the NBD-PC in the focus area using the highest laser power for 1 second, achieving an average bleach area radius of 3.75 µm. Fluorescence images were captured immediately after photobleaching.

In SOPC measurements, owing to the swift diffusion of NBD-PC, continuous fluorescence imaging was executed without interruption at a rate of 250 nm per 10 µs (pixel size/dwell time) for an area of 50 µm by 50 µm. For DPPC measurements, fluorescence images were captured every 2 minutes over a span of at least 40 minutes. The fluorescence intensity of the bleached spot was normalized to the fluorescence intensity of an unbleached reference spot, and the relative fluorescence intensity was plotted over time.

Supplementary Figure 4 illustrates a representative FRAP recovery profile of a fluorescently labeled SOPC SLB. The red curve depicts a single exponential fit to the data using the following equation:

$$y = A(1 - e^{-bt}) \qquad [1]$$

where A corresponds to the mobile fraction and b is related to the diffusion time via

$$t_{1/2} = \frac{\ln 2}{b} \qquad [2]$$

which is related to the diffusion constant by

$$D = \frac{w^2}{4t_{1/2}} \qquad [3]$$

where w is the radius of the bleaching area. Thus, we obtained a diffusion constant 0.5 $\mu m^2/s$ for SOPC with NBD-PC SLB and of 0.004 $\mu m^2/s$ for DPPC with NBD-PC. Note that the value of D for SOPC is much smaller than the previously reported value for a fluid SOPC bilayer which shows a value of D larger than 1 $\mu m^2/s$ (2). This is due to the fact that in our experiments, we did not take into account the overall photobleaching arising from the continuous scanning during the SOPC measurement.

Supplementary Note 4. Membrane mobility and E-cad mediated intermembrane adhesion

Supplementary Figure 5a and Supplementary Figure 5b show two typical time traces measured from E-cad decorated GUVs on E-cad decorated fluid SOPC SLBs. The clustering timescales (blue regions) for the fluid SOPC SLBs are in the range of 1.2 s to 1.4 s, While for the measurement of partially fluid SLBs made of DPPC and NBD-PC (Supplementary Figure 5c), the clustering timescales range from 15 s to 30 s. This observation indicates that the *cis*-clustering of E-cad is strongly dependent on membrane mobility.

Supplementary Note 5. Confirmation of the origin of consecutive ‘dip signals’

Having recognized that the dips in intensity result from the appearance of the X-dimer within the confocal volume, a question arises regarding whether the consecutive ’dip signals’ originate from the formation of X-dimer within the confocal volume or the diffusion of X-dimer. This diffusion could involve the X-dimer forming outside the confocal volume and subsequently diffusing in and out of it. To verify this, we measured the diffusion coefficient of the SLB prepared using DPPC and NBD-PC.

Previous reports indicate that these partially fluid SLB membranes, not in a full gel phase at room temperature, are not expected to be phase-separated due to the low density of Ni-NTA-DOGS used (2, 3). The diffusion coefficient of NBD-PC lipids in this SLB is estimated to be 0.004 $\mu m^2/s$ from fluorescence recovery after photobleaching (FRAP) experiments (Supplementary Figure 4), corresponding to a diffusion time of approximately 5 s. This implies that the ’dips’ are definitely not caused by diffusion, as the average waiting time of ’dip signals’ is approximately 0.05 s, much shorter than the diffusion time.

Supplementary Note 6. Determination of the high and low states

Due to thermal fluctuations in the membrane, the high state in the time trace exhibits significant noise, leading to a low signal-to-noise (SN) level. In Supplementary Figure 8b, a distribution histogram of count rates from a typical time trace reveals the challenge of completely separating the two states using a single dividing line, given the overlap between them.

To identify the high and low states, we employed a two-threshold strategy for isolating the low state from the noisy high states. In this approach, a high-threshold (proximate to the high state, Supplementary Figure 8a red line) was established to select all bursts with intensities surpassing the threshold. Subsequently, for each selected low state, the total fluorescence counts were integrated, and a low threshold was set to filter out bursts with intensities exceeding the high-threshold (Supplementary Figure 8a blue line). This two-threshold strategy ensures the inclusion of some low states with high fluorescence intensity that persist for an extended duration in the time trace.

Firstly, part of the histogram of the intensity that definitely has no low states was fitted with a Gaussian distribution:

$$I = A \exp\left[-\frac{(I - I_0)^2}{2\sigma_1^2}\right] \qquad [4]$$

where I is the intensity in counts per time bin, I_0 is the center of the Gaussian distribution, and σ_1 is the standard deviation of the photon count distribution.

Secondly, the time traces $I(t)$ were shifted to zero mean value by $I(t) - I_0$. Then, the variance σ_1 was used to set the thresholds for determining the low states and high states.

We chose the low states from the control experiment (GUV without E-cad) as N_{noise}. The number of low states extracted from the intensity trace of E-cad binding was designated as N_{signal}. We then calculated N_{noise} and N_{signal} based on the two-threshold method by setting different threshold values in units of $\sigma_1 (i \times \sigma_1)$ for these two thresholds.

Next, we calculated the ratio N_{noise}/N_{signal} as function of the values of the low and high thresholds. Supplementary Figure 8c shows the dependence of N_{noise}/N_{signal} on the

combination of the two thresholds. The red and yellow lines indicate the ratio of 10% and 5%, respectively. We found that when setting the high state at $2.5\sigma_1$ and the low state at $3\sigma_1$, the N_{noise}/N_{signal} is smaller than 2%, which means only 2% of the signal from the high state is counted as coming from the low states. We thus chose $2.5\sigma_1$ and $3\sigma_1$ as the high and low thresholds for separating the low states and high states in the fluorescence intensity trace.

Supplementary Note 7. Construction of the 2D difference histograms of waiting times

A 2D conditional histogram of waiting times for the high or low state describes the joint probability of finding pairs of waiting times ($t_{i,n}$ and $t_{i,n+1}$, where i = h or l). We also generated a correlation plot between the waiting time $t_{i,n}$ and $t_{i,n+300}$ (Supplementary Figure 10). At this larger separation, the correlation has diminished.

For the waiting time from the low state (t_l), the 2D joint histograms between the lag of 1 'low state' time and the lag of 300 'low state' times exhibit substantial changes: there is a notable excess of points distributed along the axes at larger lags ($t_{l,n}$ vs. $t_{l,n+300}$), while the scatter plots follow the inverse diagonals for two adjacent waiting times ($t_{l,n}$ vs. $t_{l,n+1}$). Conversely, for the waiting time from the high state (t_h), almost no difference was observed. The two histograms between the low states with different lags indicate a significant difference, suggesting the presence of a memory effect.

To accentuate the memory effect, 2-D difference histograms of waiting times were constructed. The 2-D difference histograms are defined as $\sigma(t_{i,n}, t_{i,n+j}) = p(t_{i,n}, t_{i,n+j}) - p(t_{i,n}) \times p(t_{i,n+j})$ for correlation analysis of one state (high state or low state), where j represents the number of cycles that separate waiting time t_n and t_{n+j}.

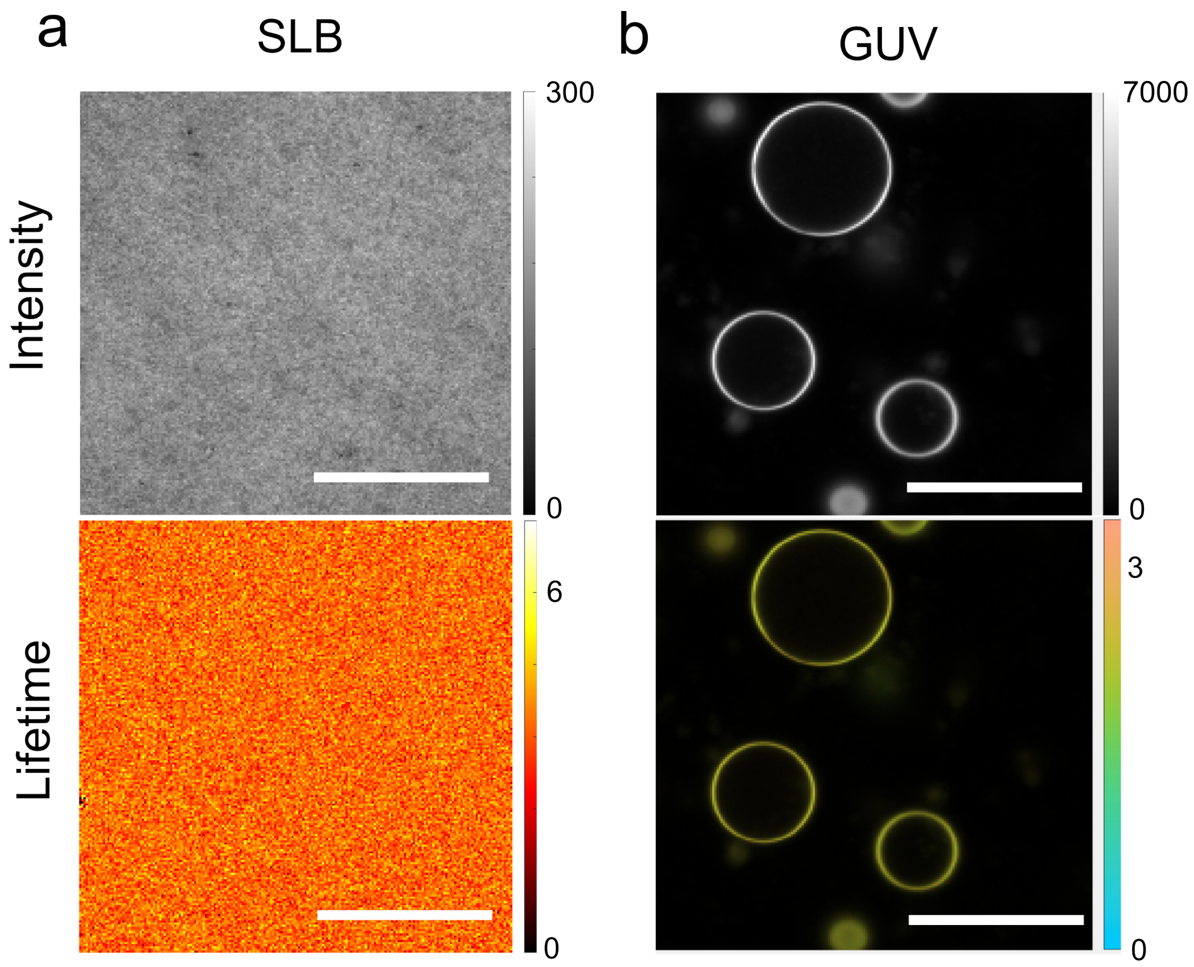

Supplementary Figure 1. Fluorescence intensity images and lifetime images of SLB and GUV. (a) Fluorescence intensity image and corresponding lifetime image of SLB. The fluorescence is from the NBD-PC, which excited via a 488 nm laser. (b) Fluorescence intensity image and corresponding lifetime image of GUV containing fluorescent lipid and Ni-NTA-DOGS molecules. No phase separation was found both in the intensity iamge and lifetime images. Scable bar: 20 μm

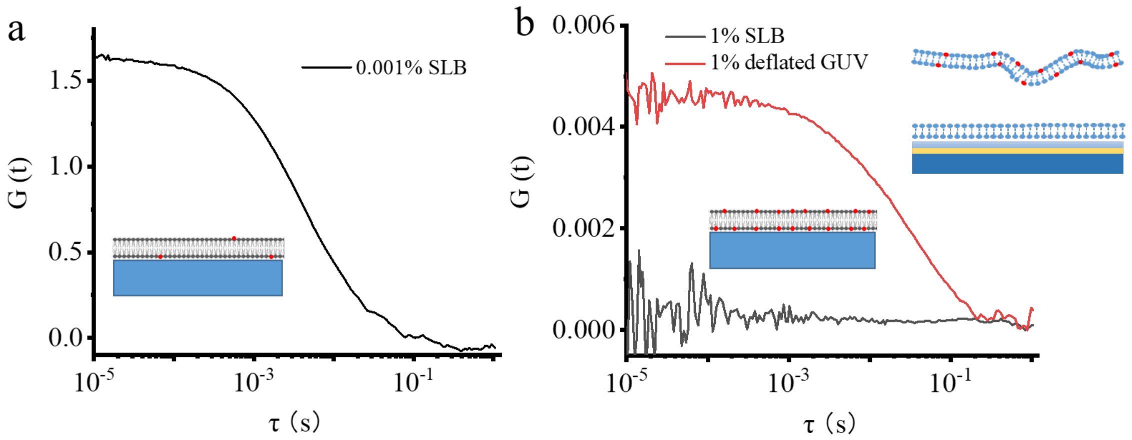

Supplementary Figure 2. ACFs measured on (a) lowly labeled SLB, (b) highly labeled SLB (black curve), and highly labeled deflated GUV (red curve).

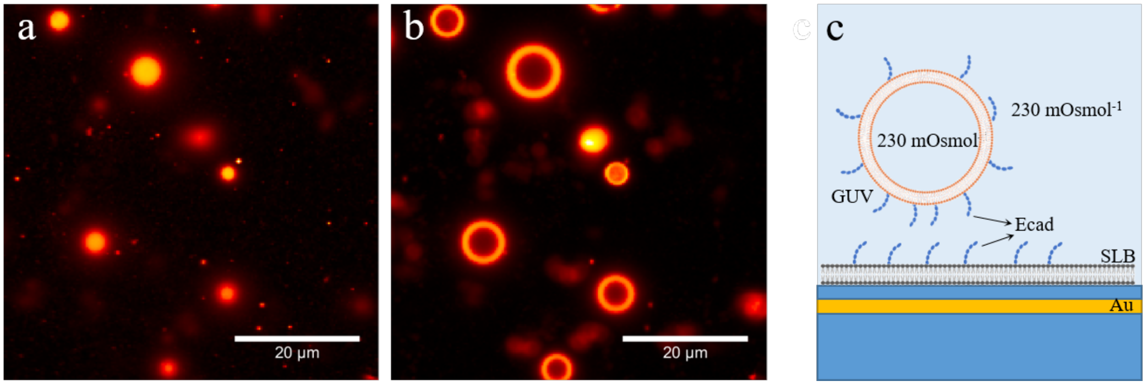

Supplementary Figure 3. Fluorescence images of E-cad-modified GUV on E-cad-modified SLB recorded (a) directly on the surface and (b) at a height of 10 μm above the surface. (c) Schematic representation of the E-cad-modified tense GUV on E-cad-modified SLB, above a gold surface with 10 nm silica spacer. Tense GUVs were prepared by immersing them into a PBS solution (230 mOsm/L), which matches the osmolality of the sucrose solution (230 mOsm/L) inside the GUV.

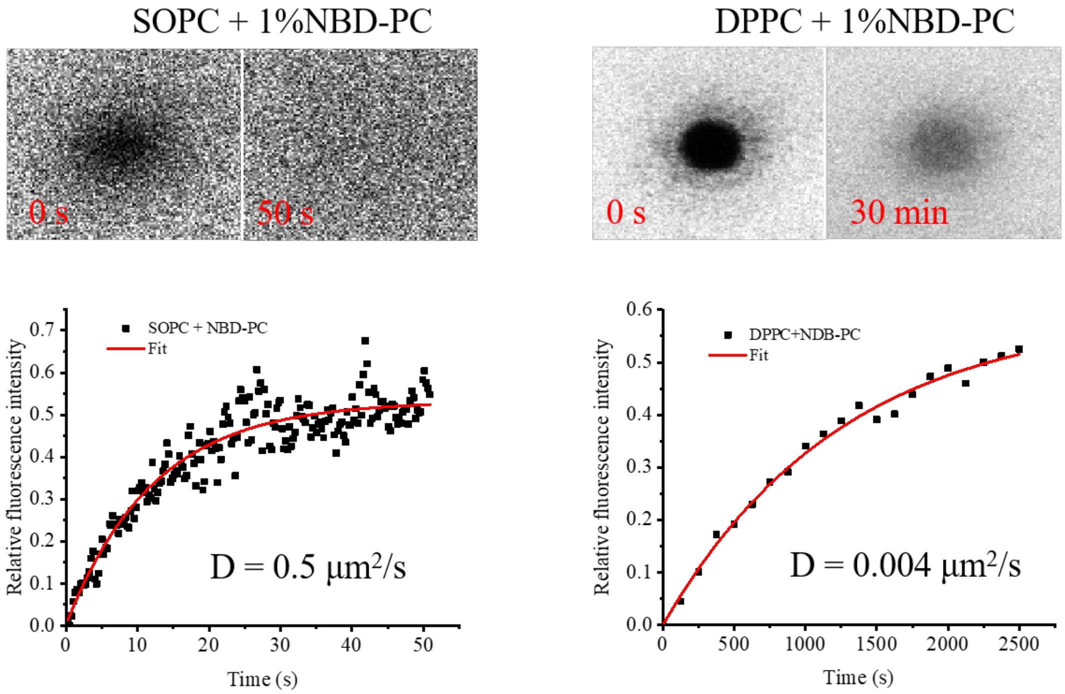

Supplementary Figure 4. FRAP analysis of SOPC (a) and DPPC (b) SLBs. Top: photobleached spot that recovers after 50 s (a) and 30 min (b). Bottom: fluorescence recovery profile after photobleaching.

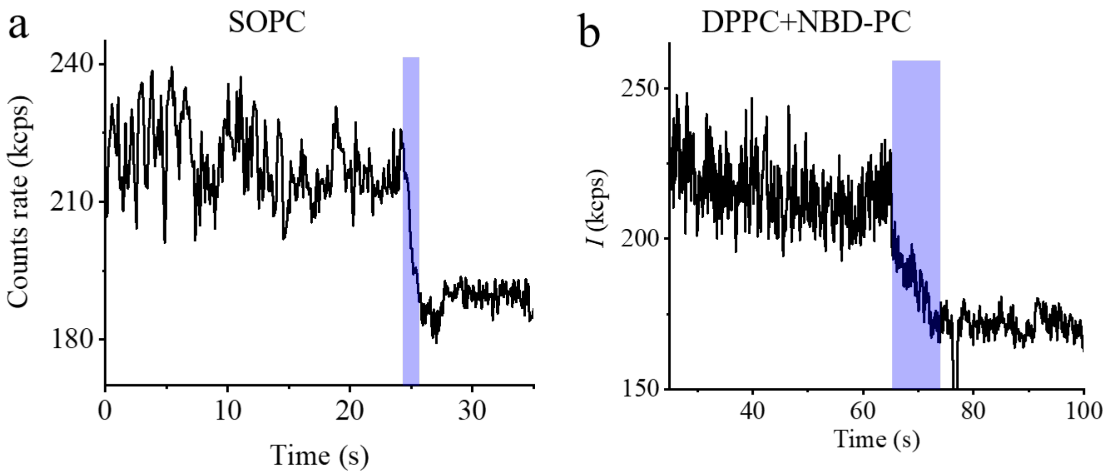

Supplementary Figure 5. Two typical fluorescence time traces of E-cad-mediated membrane adhesion with the formation of the final cic-cluster state. The trace was measured from the biomimetic system on (a) fluid SOPC SLB and (b) partially fluid SLB made of DPPC and NBD-PC.

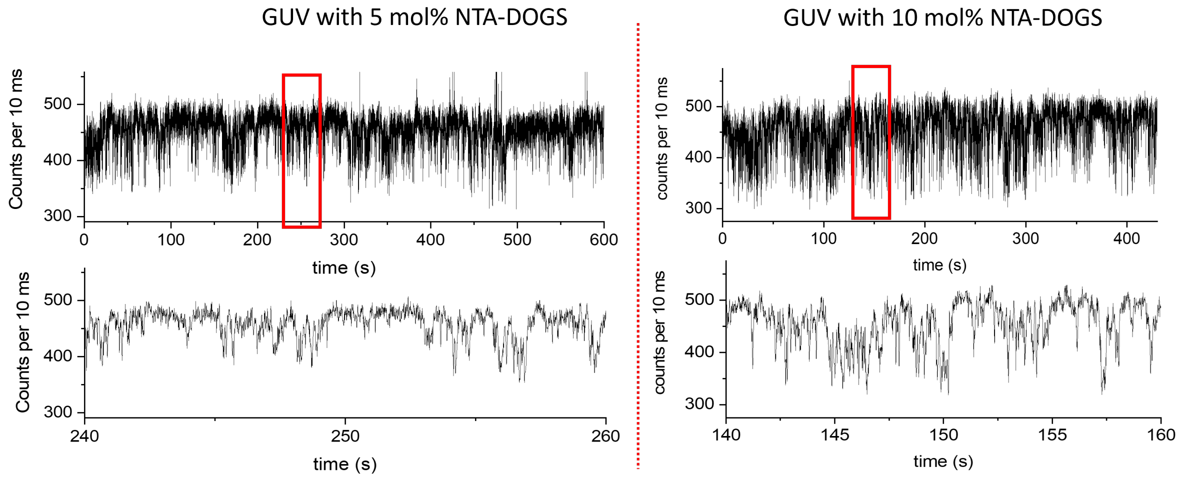

Supplementary Figure 6. Fluorescence intensity time traces obtained from GUVs with two different concentrations of E-cadherin (Ecad) on partially fluid SLBs. Enlarged plots are shown at the bottom.

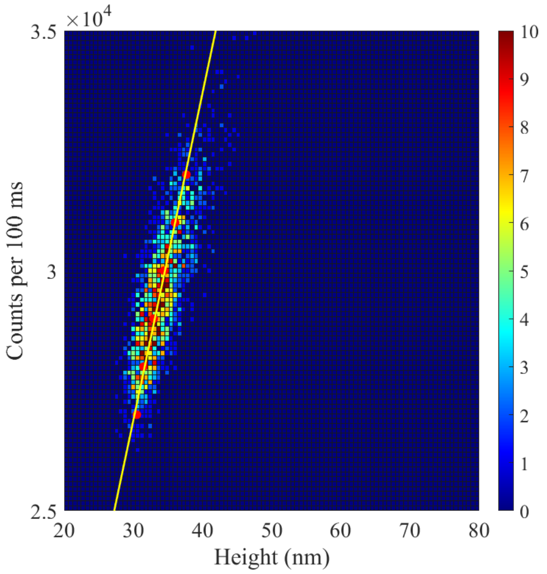

Supplementary Figure 7. A two-dimensional histogram of the fluorescence intensity and fitted lifetimes, data was measured on a sample without E-cad between GUV and SLB. The yellow line is a linear fit.

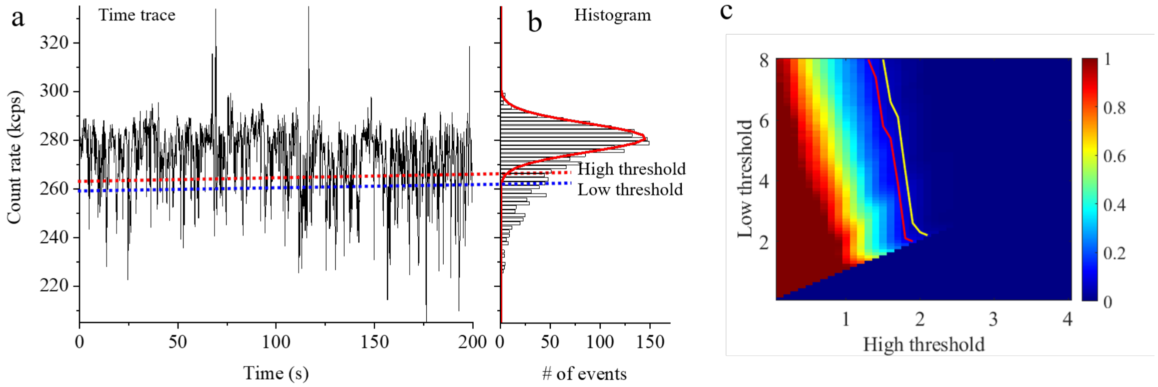

Supplementary Figure 8. (a) Fluorescence intensity trace and (b) corresponding intensity histogram. (c, d) Dependence of Nnoise/Nsignal on the combination of high and low threshold.

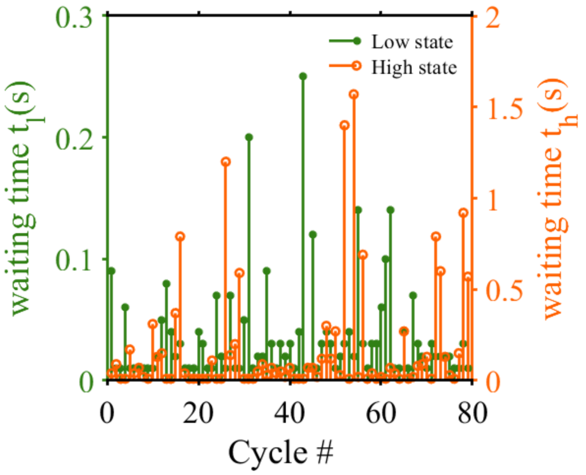

Supplementary Figure 9. Waiting time trajectories for t_l and t_h reconstructed from fluorescence time trace.

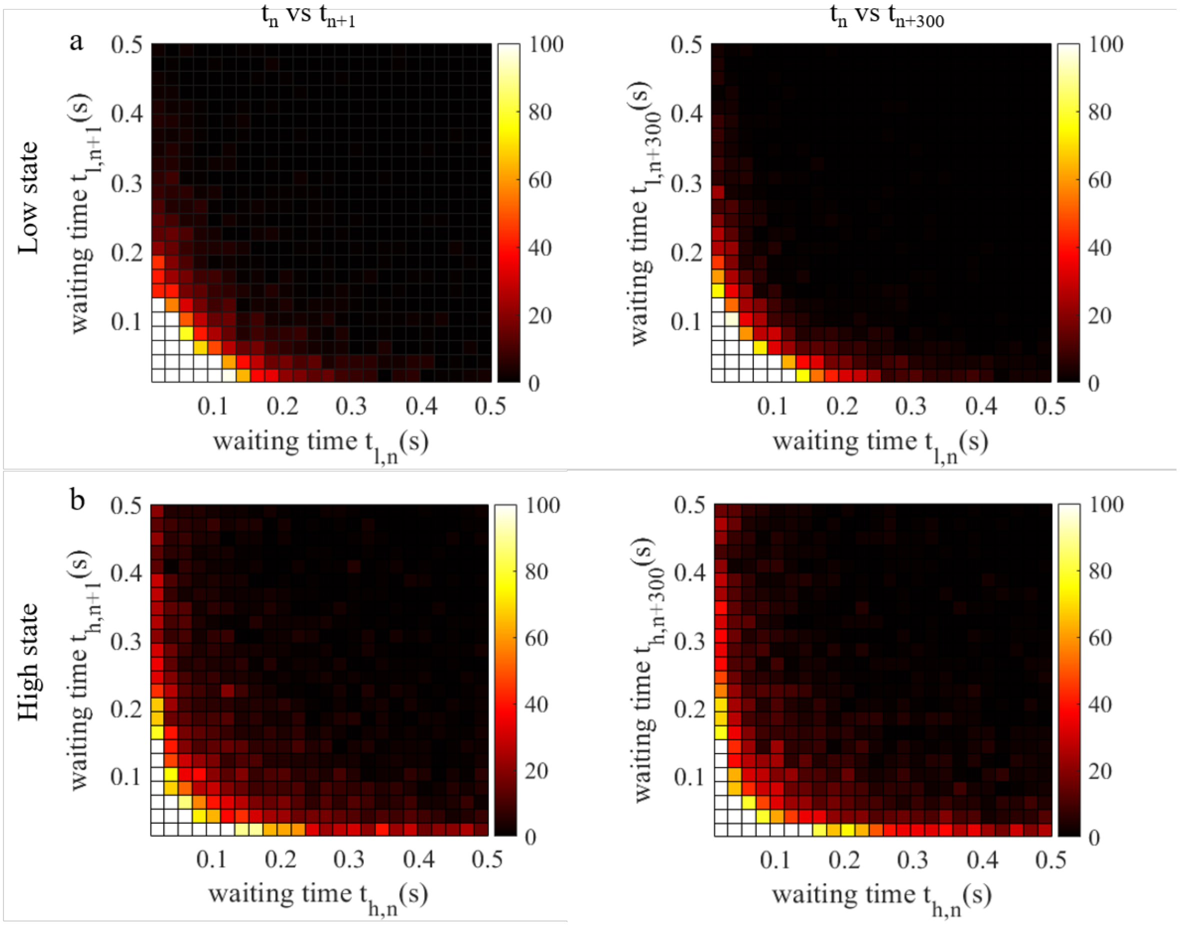

Supplementary Figure 10. 2D conditional histograms of waiting times for low state (a) and high state (b) recorded at t_n and t_{n+1} (left panel) as well as t_n and t_{n+300} (right panel).

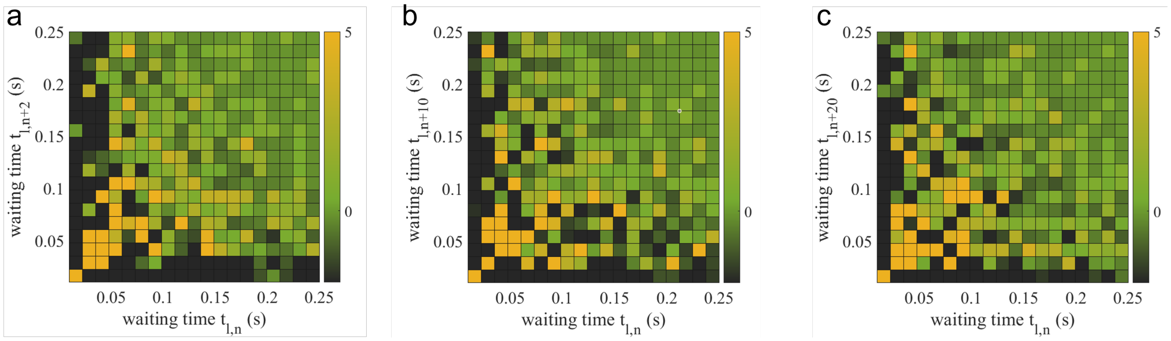

Supplementary Figure 11. 2D difference histograms of waiting times for low state, calculated for (a) t_n and t_{n+2}, (b) t_n and t_{n+10}, and (c) t_n and t_{n+20}.

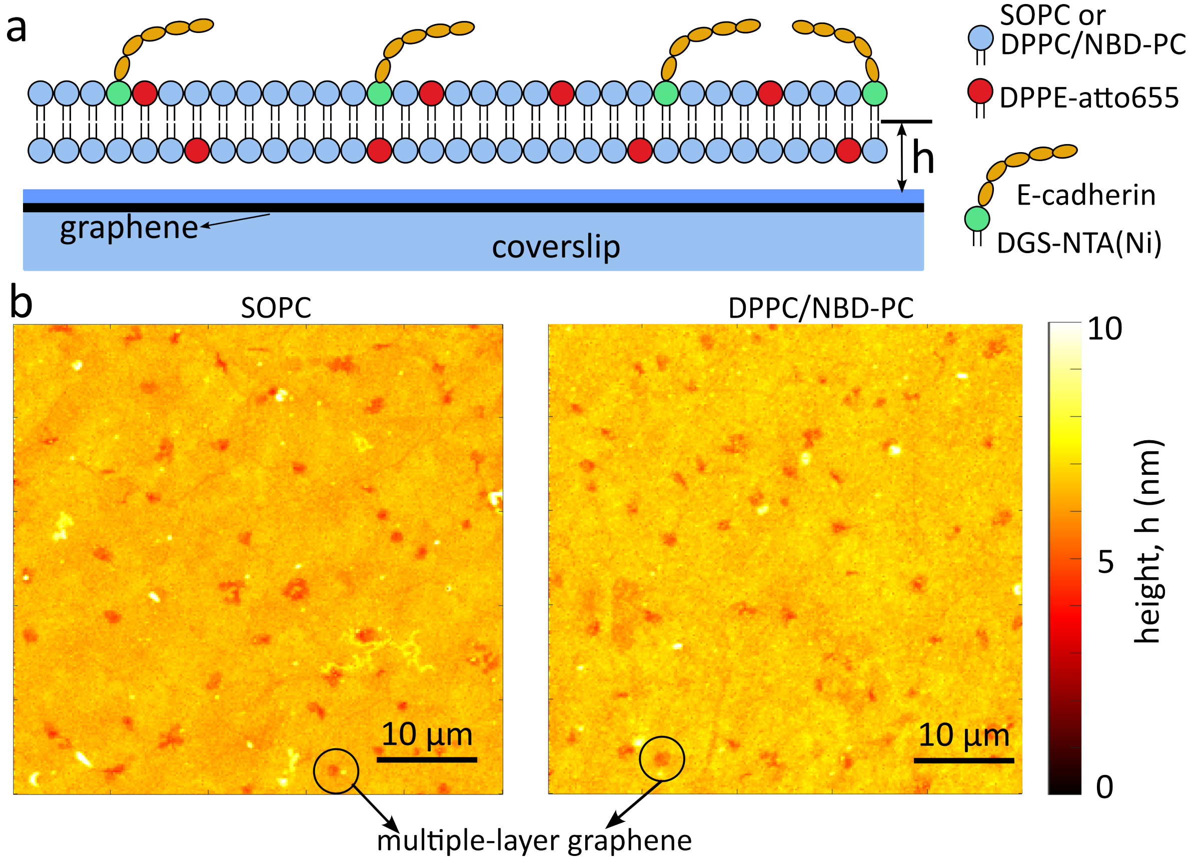

Supplementary Figure 12. Graphene-induced energy transfer (GIET) imaging for determining average SLB heights. (a) A schematic illustration of the GIET measurement setup is shown. SLBs composed of SOPC or DPPC/NBD-PC were deposited on a graphene monolayer with a 5-nm silica spacer. To measure membrane height, a small fraction of lipids was labeled at the headgroup with the fluorophore Atto655. GIET enables precise determination of the mean SLB height (h), corresponding to the average axial position of the dyes in the bottom and top leaflets, with sub-nanometer resolution. (b) Height maps are provided for both SOPC-SLB (left) and DPPC/NBD-PC-SLB (right), revealing a nearly uniform height distribution across the SLBs. Areas with reduced height are attributed to defects or regions with multiple graphene layers.

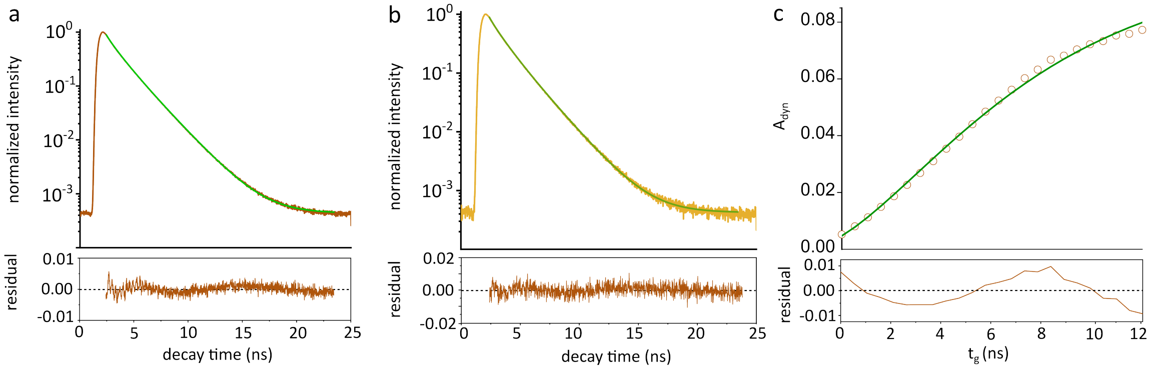

Supplementary Figure 13. (a, b) Fluorescence lifetime curves for high (a) and low (b) states. The curves were fitted using multi-exponential decay mode. Bottom is the corresponding fitting residual. (c) sg-FCS analysis and the corresponding fitting residual.

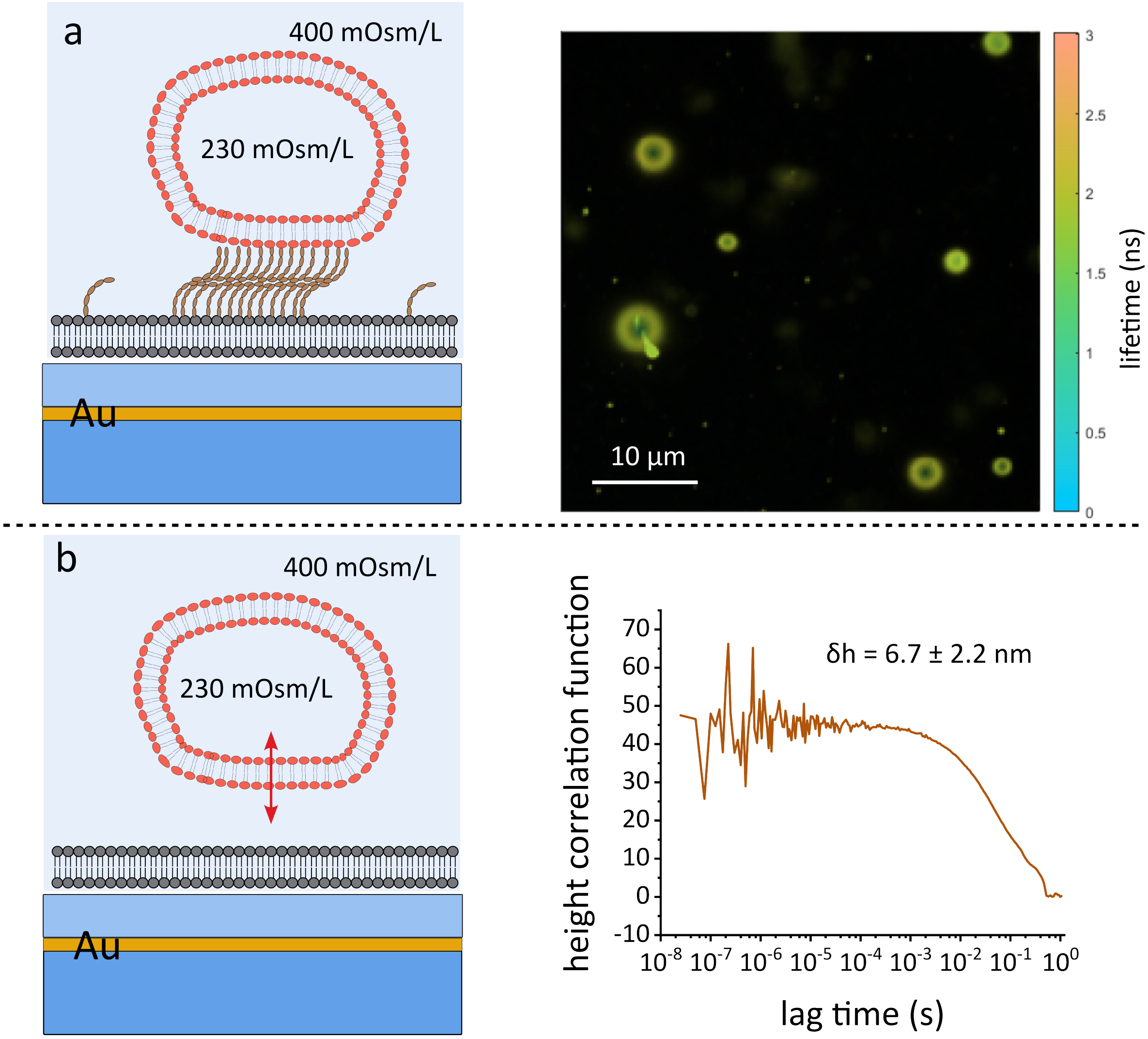

Supplementary Figure 14. (a) Left: Schematic illustration of the control measurement of the fluorescently-labeled GUVs containing E-cad on SLB. The osmolarity difference between the interior of the GUVs (230 mOsm/L, sucrose solution) and the external buffer (400 mOsm/L, phosphate-buffered saline, PBS, mixed with 0.75 mM $CaCl_2$ solution) is 170 mOsm/L. Right: Corresponding FLIM image captured 10 minutes after incubating the GUVs in the chamber. Multiple areas within the chamber were examined, and no GUVs exhibited the X-dimer dynamic transition (as described in the second scenario in the main text). (b) Left: Schematic illustration of the control measurement of the fluorescently-labeled GUVs without E-cad on SLB. The bottom membrane of GUV undergoes fluctuation. Right: The calculated height correlation function, indicating the membrane fluctuation amplitude of 6.7 $\pm$ 2.2 nm.

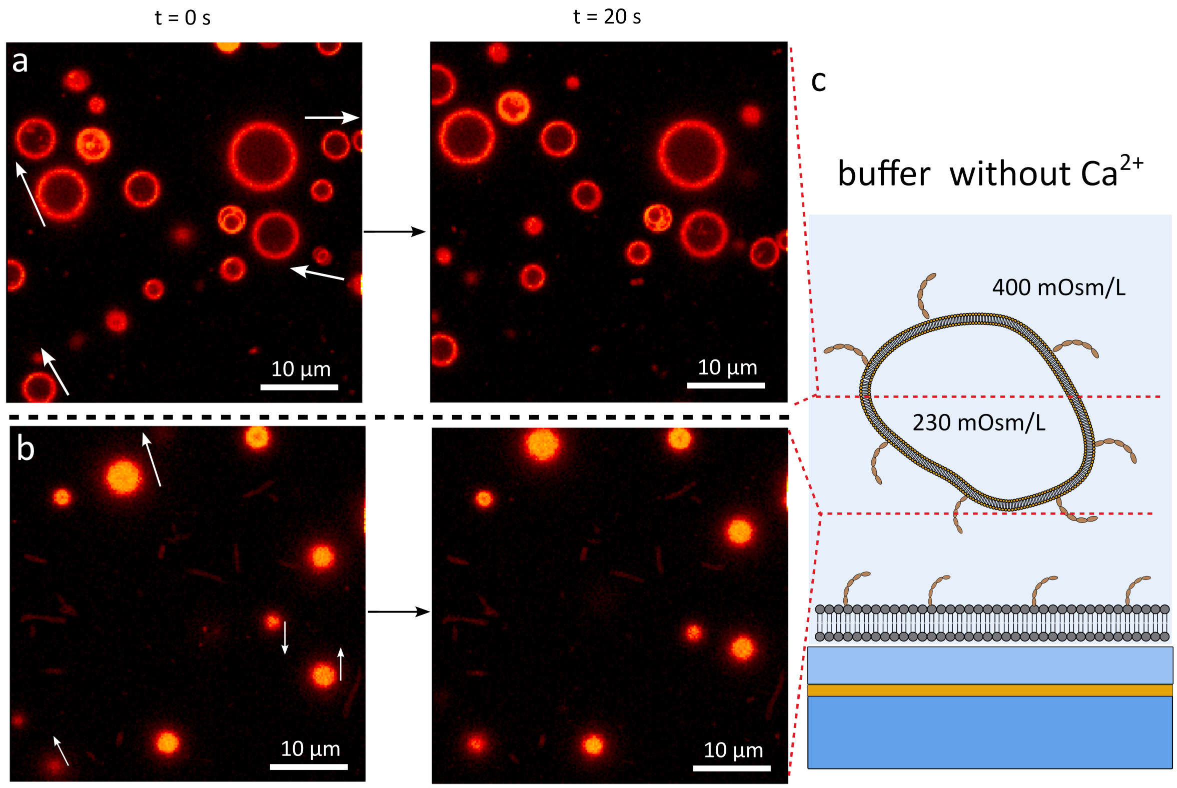

Supplementary Figure 15. Fluorescence images of E-cad-modified GUV on E-cad-modified SLB recorded at two different focal planes: (a) 10 µm above the surface and (b) directly at the surface. The images on the right were recorded 20 seconds after the images on the left. White arrows indicate the direction of vesicle movement. (c) Schematic representation of the E-cad-modified deflated GUV on E-cad-modified SLB, above a gold surface with 10 nm silica spacer. GUVs were prepared by immersing them into a PBS solution without Ca^{2+} (400 mOsm/L).

Supplementary References

1. C Monzel, et al., Measuring fast stochastic displacements of bio-membranes with dynamic optical displacement spectroscopy. *Nat. commun.* **6**, 1–8 (2015).
2. KH Biswas, et al., E-cadherin junction formation involves an active kinetic nucleation process. *Proc. Natl. Acad. Sci.* **112**, 10932–10937 (2015).
3. LM Loura, F Fernandes, A Fernandes, JP Ramalho, Effects of fluorescent probe nbd-pc on the structure, dynamics and phase transition of dppc. a molecular dynamics and differential scanning calorimetry study. *Biochimica et Biophys. Acta (BBA)-Biomembranes* **1778**, 491–501 (2008).
