# Supplementary material for: Observation of E-cadherin adherens junction dynamics with metal-induced energy transfer imaging and spectroscopy
Source: Commun Biol. 2024 Nov 30;7:1596. doi: 10.1038/s42003-024-07281-4 (PMC11606943; doi:10.1038/s42003-024-07281-4)
Supplement: Supplementary file 3 — Description of Additional Supplementary File [file 42003_2024_7281_MOESM3_ESM.pdf]

Description of additional supplementary file

File name: Supplementary Move 1
Description: A movie shows a process of E-cadmodified GUV binds to an E-cad-modified SLB and finally adheres to the SLB due to the formation of the cis-clustered state of the E-cads.

File name: Supplementary Move 2
Description: A movie shows the fluctuation of an E-cad-modified GUV above an E-cad-modified SLB. Notably, adhesion does not occur due to the absence of the cis-clustered state formation.
